# Being Heard: A Qualitative Study of Lithuanian Health Care Professionals’ Perceptions of Dignity at the End-of-Life

**DOI:** 10.3390/medicina57121318

**Published:** 2021-12-01

**Authors:** Rūta Butkevičienė, Jolanta Kuznecovienė, David Harrison, Eimantas Peičius, Gvidas Urbonas, Kristina Astromskė, Ramunė Kalėdienė

**Affiliations:** 1 Department of Bioethics, Lithuanian University of Health Sciences, Tilžės Str. 18, LT-47181 Kaunas, Lithuania; jolanta.kuznecoviene@lsmuni.lt (J.K.); wdavidharrison@mac.com (D.H.); eimantas.peicius@lsmuni.lt (E.P.); gvidas.urbonas@lsmuni.lt (G.U.); 2 Department of Health Management, Lithuanian University of Health Sciences, Tilžės Str. 18, LT-47181 Kaunas, Lithuania; kristina.astromske@lsmuni.lt (K.A.); ramune.kalediene@lsmuni.lt (R.K.)

**Keywords:** dignity, end-of-life, qualitative research, phenomenology, dying patient, Lithuania

## Abstract

*Background:* The literature on professionals’ perceptions of dignity at the end-of-life (EOL) shows that there is a need for studies set in different cultural contexts. Lithuania represents one of these little-studied contexts. The aim of this study is to understand professionals’ attitudes, experiences, and suggestions concerning EOL dignity to provide knowledge upon which efforts to improve EOL care can be grounded. The research questions are “How do Lithuanian health care professionals understand the essence of dignity at the end-of-life of terminally ill patients?” and “How do they believe that dignity at the EOL can be enhanced?”. *Materials and Methods:* The study was exploratory and descriptive. It employed an interpretive phenomenological method to understand the essence of the phenomenon. Lightly structured interviews were conducted with professionals who had EOL experience, primarily with elderly and late middle-aged patients. from medicine, nursing, social work, and spiritual services. The interviews were primarily conducted by audiovisual means due to pandemic restrictions. Using a constant comparative method, the research team systematically codified text and developed themes by consensus after numerous analytic data iterations. *Results:* Four primary themes about EOL dignity were identified: Physical Comfort, Place of Care and Death, Effects of Death as a Taboo Topic, and Social Relations and Communication. A fifth, overarching theme, Being Heard, included elements of the primary themes and was identified as a key component or essence of dignity at the EOL. *Conclusions:* Patient dignity is both a human right and a constitutional right in Lithuania, but in many settings, it remains an aspiration rather than a reality. Being Heard is embedded in internationally recognized patient-centered models of EOL care. Hearing and acknowledging individuals who are dying is a specific skill, especially with elderly patients. Building the question “Is this patient being heard?” into practice protocols and conventions would be a step toward enhancing dignity at the EOL.

## 1. Introduction

All studies concerning dignity at the end-of-life (EOL) are set in the context of country and culture. In this study of perceptions of dignity in the final stages of life, Lithuanian living conditions and health are thus important to consider. Quality of life has been studied extensively [1] if not uniformly. International comparisons abound. For example, the widely used Numbeo scale [1,2] integrates eight factors, including health care into a single rating. Numbeo rates Lithuania as the country with the 19th highest quality of life (scale score 160.29), just below Spain (163.48) and the United States (163.60), and just above Portugal (159.83) and the Czech Republic (157.49). On the Numbeo subscale entitled Health Care Index, Lithuania ranks 28th of the 83 countries studied. 

But this picture stands in contrast to data not directly about health care systems, but about the prevalence of two common causes of death, cancer, and heart disease. Both general categories of disease are likely to involve treatment in medical institutions. Lithuania’s rate of cancer is 16 percent higher than Europe as a whole (184 per 100,000 versus 159 per 100,000 in 2018) [3]. Lithuania’s ischaemic heart disease rate has declined since the crises of gaining independence in 1990, when the rate was 428 cases per 100,000 people. However, the Lithuanian rate in 2018 was 276 per 100,000, still well above the comprehensive rate of 67 per 100,000 for Europe [4]. Since many of these individuals will die of their conditions, these rates have important implications for the health care system and how services are provided. These data provide hints about the demands for care at the EOL. Our concern in this study is one element of this heavy demand for care, namely EOL dignity. Unlike quality of life, quality of death has not been studied extensively, and this study undertakes an exploration of the meaning of dignity to those professionals who provide care in these circumstances.

Guo and Jacelon documented core elements that research has shown constitute dignity at the end-of-life (EOL). In “the first integrative review to synthesize international evidence regarding the meaning of, and common aspects of, dignity in end-of-life care”, the researchers highlighted the need for further inquiry “to evaluate the meaning of dying with dignity across cultures” [5] (p. 939). Researchers have begun to address these needs, but given the breadth of the subject, findings are only beginning to form a body of knowledge that spans environments. Following this progression of research, the current exploratory, interpretive phenomenological study addresses two significant questions as experienced by Lithuanian medical professionals. How do Lithuanian health care professionals understand the essence of dignity at the EOL? How do they believe that dignity at the EOL can be enhanced? The aim of this study is to understand professionals’ attitudes of mind, experiences, and suggestions to provide knowledge upon which efforts to improve EOL care can be grounded.

In a series of studies first reported in the early 2000s, Chochinov and colleagues pioneered in the conceptualisation of dignity at the EOL in the context of medical settings. The team, based in Canada, integrated quantitative and qualitative methods with clinical experience to develop a model for definition, promotion, and preservation of dignity [6,7,8,9,10,11,12]. Noting the limited attention to the elderly in previous research, they included this population in their studies [10]. The framework for understanding and promoting dignity included patient, spouse, and physician perspectives. The model presented in the studies includes illness-related concerns (e.g., level of independence, symptom distress), dignity conserving perspectives (e.g., hope, role, generativity, pride), dignity conserving practices (e.g., maintaining normalcy, spiritual comfort), and an inventory of components of social dignity (e.g., privacy, support, burden, tenor of care). The Chochinov approach of discovering and listing essential components of dignity and ways that it can be enhanced has been a cornerstone in much of the subsequent research on the topic.

The study of dignity at the EOL (EOL) has progressed in the last 20 years. Guo and Jacelon’s major review article has become a foundation for understanding dignity as a component of EOL experiences. The authors reviewed 52 studies which met the criteria of their study and found that the majority of research conducted on the topic was from North America, Western Europe, and the United Kingdom. Other research included samples from Australia, Ethiopia, India, Kenya, and Romania. Numerous studies included professional health care providers [5]. This distribution of locations and foci led the authors to the conclusions that more research was needed in different cultural contexts and with families.

Elements of a definition of dying with dignity (not to be confused with the same term that is sometimes used in other contexts to connote physician-assisted suicide or euthanasia) can be found in these research articles. Some authors state or emphasize one element, while others accentuate different components. Guo and Jacelon synthesized a definition of dignity from the research that they reviewed. Their definition is as follows: dying with dignity is a basic human right; it is a subjective experience and also a value influenced by others; it signifies a dying process with the following characteristics: dying with minimal symptom distress and limited invasive treatment, being human and being self, maintaining autonomy and independence to the greatest extent, achieving existential and spiritual goals, having self-respect and being respected by others, having privacy, maintaining meaningful relationships with significant others, and receiving dignified care in a calm and safe environment [5].

The Guo and Jacelon synthesis shows the difficulty of specifying a circumscribed definition of dying with dignity, even though it is universally understood as a desirable state. The definition includes components of a right, an experience, a value, and a process with a set of characteristics. This array reflects the complexity and variability of dying with dignity. The authors raise some central questions about the specific constellation of these factors, including how much of the observed variability is due to culture and location. 

Pringle, et al. conducted a second significant review of studies on the topic of dignity as understood by individuals who were experiencing EOL care, professionals, and family members. The researchers found that patient and family experiences with organized care programs tended to be more favourable than those of families that experienced conventional care. Pringle et al. joined Guo and Jacelon in the conclusion that hospital environments pose a threat to dignity [13].

Recently Silva et al. published a review article on the understanding of EOL dignity and its implications in Middle Eastern countries. Fourteen of the 16 articles focused on Iran. They noted that of the 16 relevant articles, 12 focused on patient perceptions, one of them discussed dignity from the family caregivers’ perspective, and the remaining articles analyzed perceptions reported by nurses, physicians, and other hospital staff members. Research participants reported the following preconditions for dignity at the EOL: gentle communication with a dialogue that preserves hope instead of blunt truth-telling, abundance characterized by accessibility to medical supplies and financial stability, family support where relatives deliver major assistance in care, physical fitness, reliable health care, and social justice that endorses equal care to all. The authors also noted that although the results were compatible with the existing evidence from the Dignity Model ascertaining that dignity is socially mediated and influenced by interactions and physical fitness. Nevertheless, the findings highlight that patient dignity is also shaped by the socio-political, cultural, and economic conditions of the country, where family support, gentle communication and accessible health care are essential elements [14] (p. 112).

Studies of professionals’ perspectives on EOL dignity have tended to rely on measures or scales concerning facets of dignity. Understanding the *a priori* assumptions implicit in these scales is important especially in cross-cultural research where there may be very different understandings of professional practice to enhance EOL and dignity itself. The assumption that dignity is primarily related (inversely) to distress and that information about patients is likely to improve the situation are evident in a study by Bovero et al. [15]. The researchers compared Italian nurse assistants, nurses, physicians, and psychologists to determine their perspectives on the sources of patient dignity using a modification of the Patient Dignity Inventory developed by Chochinov et al. [9]. Bovero et al. summarized the results as follows: 

Considering their importance for the HCPs [health care professionals], the psychological area was ranked first and the physical area second for all the HCP groups, except for the psychologists for which it was the opposite. All groups evaluated the social and existential areas as less important for a hypothesized clinical intervention on the patient’s dignity than the psychological and physical areas [15] (p. 1188).

Bovero et al. also interviewed subjects to order the to learn whether they had participated in training on EOL dignity, whether and what “information on the patient” they needed to develop an “appropriate intervention on the patient’s dignity”. The physicians with some training “evaluated the dignity-related psychological, existential, and spiritual distress as more salient than physicians that did not attend such training.” [16] (p. 6). The implication of these studies is that dignity is somehow related to, created through, or enhanced by a process of professionals intervening or treating people at the end of their lives, although self-selection may also be involved.

Overwhelmingly, the studies have been framed from the viewpoint of research professionals. This may lead to the generalisation of methods and particularly scales that may or may not be valid relevant in different linguistic and cultural contexts. Frequently studies have been based on the tacit assumption that dignity can be enhanced by professional behaviors. Sometimes this results in framing the dying individual as the object of dignity-enhancement behavior on the part of the professionals rather than as a relational construct. With the exception of some of the Chochinov studies, relatively little has been documented about interpersonal processes in maintaining or enhancing dignity, regardless of how it has been defined.

### The Lithuanian Context

The studies cited above show considerable progress in the study of dignity in the context of EOL care. However, the existing body of research only slightly addresses a number of critical questions. Most important for the current study are questions about how local cultural context, professional views, and both informal and clinical practices have contributed to or detracted from dignity. Most significant for the current study is the fact that dignity at the EOL has been studied very little in Lithuania.

Lithuania is fast entering the European mainstream of expectations and technological possibilities concerning EOL care, which suggests that this could be a time of significant development in the understanding of dignity. However, the terrains of both practice and research are only now taking shape. Innovations have been difficult, as illustrated in a series of studies documenting attempts to begin innovative interdisciplinary integrated care projects [17,18,19]. Most settings have not yet fully adopted the orienting principle of patient-centered care, despite aspirational legislation and policies intended to promote or mandate the idea. For example, Article 3 (point 6) of the Lithuanian Law on the Rights of Patients and Compensation of the Damage to their Health simply states that “Patients have the right to be cared for and to die with dignity” [20].

Two Lithuanian studies conducted by nurse researchers are noteworthy, if not directly concerned with perceptions of dignity. Blaževičienė et al. studied a sample of 239 Lithuanian oncology nurses to determine their perceived role and obstacles in providing end-of-life care. Participants worked in surgical, therapeutic, and intensive care settings in two university hospitals. While the focus of the study was on attitudes about, and barriers to, providing palliative end-of-life care rather than explicitly addressing perceptions of patient dignity, the authors indirectly defined dignity in terms of meeting spiritual needs and pain control:

According to the study, RNs working in the three different profiles emphasized safe and effective care for patients at the EOL. RNs also emphasized the importance of meeting the patient’s spiritual needs in EOL care, i.e., the patient should have the right to a dignified and painless death [21] (p. 3).

Working from the perspective of nursing, the authors found the following obstacles to be most important in providing palliative care:

Major obstacles in providing care included the nurse’s opinion that immediate patient care was not valued, lack of nursing knowledge on how to treat the patient’s grieving family, and physicians who avoid conversations with the patient and family members about diagnoses and prospects [21] (p. 1). 

Blaževičienė et al. subsequently studied Lithuanian nurses’ opinions about obstacles and supportive factors, as well as opinions of the role of nurses in palliative end-of-life care. The authors stated that “Palliative care is fundamental to health and human dignity and is a basic human right” [22] (p. 1), thus affirming the link between care and dignity. The team used a Lithuanian adaptation of a standardized instrument in a large survey (N = 1055) covering nurses employed at seven comprehensive hospitals. Spiritual needs of patients were emphasized by participants as the primary area of need for patients. Using the authors’ link between palliative care and dignity, spiritual needs might logically be thought of as a significant element of dignity. Three major obstacles were as follows:

Three major obstacles were identified by more than 80% of all categorized respondents: Nurses have to deal with angry patient’s family members; The patient’s relatives having inadequate understanding of the situation interfere with the nurses’ duties, and usually there is not time for conversations with patients about their wishes concerning the end-of-life issues/decisions [22] (p. 4).

With these significant studies as background, the current research looks more specifically at a phenomenological perspective in which providers discussed the meaning of, barriers to, and ways to enhance dignity. The study reported here is a component of the research project “Ensuring dignified EOL of terminally ill patients in Lithuania: conceptions, expectations, possibilities and obstacles” (funded by Lithuanian Science Foundation, grant number S-GEV- 20-2). The current study aims constitute a component of the overall project aims, which are to explore and evaluate concepts, needs, expectations, barriers, and inequalities related to dignified EOL care in Lithuania. A desired outcome is a set of guidelines to enable authorities, health professionals, and social care workers to use as they protect and enhance the dignity of terminally ill patients. The current study builds on earlier theoretical and qualitative components of the project [23,24,25].

## 2. Materials and Methods

### 2.1. Assumptions

The study began with some informed, a priori conceptual ideas about what dignity might mean. These assumptions were drawn from sources including those already cited and from the practical experience of the researchers as social workers and social scientists. The researchers worked to clarify their starting point in terms of knowledge and assumptions. The central organizing a priori assumption guiding the current study was that understanding dignity (subjective well-being) means not just valuing a person, but also the way that people experience valuing and being valued. Dignity is both personal and relational. The interacting experience of values through relationships includes cultural, religious, familial, professional elements. Since many people at the EOL are aged, intergenerational elements are especially important. All of these components presume that communication of a sense of worth leads to subjective well-being. The broad orienting framework that the researchers adopted as representative of their outlook and orientation is well summarized by Mattson and Clark:

Dignity is variously viewed as an antecedent, a consequence, a value, a principle, and an experience, from philosophical, legal, pragmatic, psychological, behavioral, and cultural perspectives [26] (p. 303).

Mattson and Clark went on to specify their definition, in which human dignity is “defined as a subjective experience of well-being contingent on the collective sum of (inter-)individual experiences of values” [26] (p. 316).

### 2.2. Research Approach

To study used a phenomenological orientation and a thematic analysis analytic approach to qualitative inquiry. It was intended to elicit an open-ended understanding of the meaning of dignity at the EOL as experienced by professionals, in contrast to previous studies that have used checklists of pre-determined items and variables. Assessing the reliability and validity of a qualitative study is different from assessing them in a quantitative study. While some checklists of criteria for qualitative studies are sometimes used to assess qualitative studies, these typically refer to procedures and methodology derived from a positivistic application of standards. The Robert Wood Johnson Foundation’s Qualitative Research Guidelines Project Evaluative Criteria section emphasizes that benchmarks for quality should correspond to the philosophical stance and the aims of the researchers [27]. Thus, constructs such as randomisation, convergent and discriminant validity of measurement scales, and statistical significance testing would be inappropriate in a study that does not use formal measurements. Using pre-existing or original scales would be inappropriate in this type of phenomenological inquiry. 

There have been many delineations of the characteristics of quality in a qualitative study. Many published assessments were reviewed and expanded upon by Whittemore et al. [28]. Almost all incorporate the seminal work of Lincoln and Guba [29] that, as Cypress stated, “established these four criteria as benchmarks for quality credibility, transferability, dependability, and confirmability” [30] (p. 257). Cypress summarized the nature of each of these benchmarks. Credibility is the accurate and truthful depiction of a participant’s lived experience. Transferability refers to clarity about the phenomenon and participants in the study, presentation of data and findings that others can compare to other relevant phenomena and situations. Dependability is similar to reliability as understood in quantitative research, and it involves examining the data critically from several viewpoints for convergence and divergence of coding and derived themes, particularly when multiple analysts compare their intermediate findings. Confirmability refers to the ability of the study to follow a logic that can be understood and assessed by others, often in the form of reviewing transcripts and ongoing logs of the analysis process. The current study incorporates each of these criteria in a process that is open to external examination of data, intermediate work, and discussion of results. The process used is detailed below in the Data Analysis sections.

Little has been reported about dignity and care at the end-of-life in Lithuania except through studies that requested responses to items with pre-determined response options. A different approach was taken in the current study. In order to broaden and deepen understanding, the current research team employed an inductive, phenomenological approach as articulated by Pietkiewicz and Smith [31] for the detailed study of experiences as perceived by people in a particular type of circumstance, in this case end-of-life care, primarily with elderly and advanced middle-aged patients. The approach was chosen in order to learn about dimensions of dignity from the points of view of the people providing care in formal professional capacities. An in-depth interview approach was chosen to allow participants to develop their narratives to encompass experiences and recommendations. From their accounts and suggestions, the picture might be broadened and made more useful as a guide to improved well-being and care. Qualitative methods were chosen for gaining insight into underexplored phenomena [32] such as these. 

The research team developed a simple interview guide composed of six stimulus questions (see Supplementary File S1: Interview Guide). Originally, there were many more questions, but after preliminary trials with volunteers, the guide was simplified and focused. The six questions covered professionals’ perceptions of patient and family needs, difficulties encountered in meeting patient needs, and barriers to ensuring a dignified EOL The central and most productive questions were “How do you personally understand dignified end-of-life?” and “What factors, according to your professional experience, ensure dignity at the end-of-life?”. These were followed with a variety of probes and restatements by the researcher to enhance their understanding. In this type of research, validity of responses is based on the participant’s self-report of how they want their perception of the phenomena being asked about. The responses, including elaborations in response to probes and interviewer requests for verification that they understand the responses are appropriately taken at face value.

The PhD-level interviewers held credentials in social work and sociology and had sufficient experience with qualitative studies and topics related to end-of-life phenomena to be aware of and open to the range of possible experiences that might be shared by participants to understand the meaning of the “subjective experience of well-being” to the study participants. One element of being open is awareness that meaning often comes by specifying the untoward experience of its opposite.

### 2.3. Sample

The study sample focused on professionals who had direct experience with providing end-of-life care. Participants were chosen to constitute the sample in order to develop a triangulated understanding combining the experiences of multiple professions. Consistent with phenomenological guidelines, the sample was chosen deliberately in order to ensure that participants would have ideas and experiences to share based on their experience. There was no pre-determined quota for sample size, and sampling continued until categories and themes became repetitive. Sampling ceased when the essential emic understanding of the data was reached and agreed upon by the research team reviewing the data, and it was further agreed that additional interviews would probably yield few additional conceptual categories. 

To gain the participation of individuals who could provide relevant data, the research interviewers engaged participants in two ways, using purposive and snowball principles as appropriate in a qualitative phenomenological study [31,33,34]. The first method of engagement was appeals issued through relevant organisations. Professionals were initially recruited by referral through members of the Society of Palliative Medicine. The researchers directly engaged potential participants through personal contacts, and similarly participants who had been recruited were asked to suggest additional individuals who they thought had relevant perspectives to offer.

Prior to the study the research project was approved by Kaunas Regional Committee of Ethics of Biomedical Research. The aim of the study was explained personally to each participant including the meaning of terms, possible inconveniences involved in participation, withdrawal rights, and a guarantee of confidentiality and data protection. Each informant’s written informed consent was obtained, or in the case of interviews that had to be conducted using audio-visual software because of highly restricted pandemic conditions, recordings were made and recorded to document that each participant had understood and orally agreed to the research process.

During the first contact with potential informants, they were offered two interview media options, in-person or audio-video. Three professionals preferred in-person interview. The rest of the interviews were conducted using audio-video software including Zoom and Microsoft Teams. Audio-video choice was influenced by several reasons as following: security restrictions due to the COVID-19 situation, convenience, and flexibility (possibility to agree on a time change in case of unforeseen obstacles), as well as IT skills acquired during quarantine. Interviews lasted between 45 and 95 min.

### 2.4. Participants

The 21 professionals in the study cared for individuals at the end of their lives. The participants included physicians (6), nurses (7), nurse assistants (1), social workers (2), social work assistants (2), psychologist (1), and spiritual counselor (2). One additional participant was a manager and direct care provider at an end-of-life facility who did not have a professional credential. Four of the physicians and one of the nurses held administrative as well as clinical appointments. The sample included professionals providing inpatient services, home care services, or both. Their patients were overwhelmingly aged or middle-aged individuals.

### 2.5. Data Analysis

The interpretive phenomenological analysis method (IPA) was used. As a technique for qualitative data analysis the IPA has been succinctly described by Smith et al. [35] and Pietkiewicz and Smith [31]. However, as both sets of authors emphasize, guidelines should not be accepted as a final, prescriptive analytical instrument or methodology for IPA, but rather as a framework which could be adapted by each researcher. The basic process of IPA means transition from description to interpretation to understand experiences as understood by the informant. The data analysis process does not pretend to ensure objectivity through the use of detailed, rigid procedures. 

The project team researchers examined empirical interview data by applying an open-coding technique in order to minimize interpretations coming from literature and other *a priori* sources [36]. The aim was to identify themes, ideas, experiences, and meanings which repeatedly appeared in the informants’ narratives. Consistent with IPA, the research team-based data analysis on inductive methodology, with the aim of moving from the specific to a conceptual or theoretical framework based on findings derived from interview data. Both the explicit (semantic) and latent contents of narratives were considered in data analysis. This analysis allowed for synthesis and interpretation of assumptions, commonalities, and contradictions in the data.

The researchers moved through a progression of stages. First, interview data was recorded and transcribed shortly after each interview was conducted. Soon after the interview each was transcribed, and the two interviewing researchers started with a familiarisation procedure. In this phase, the researchers read all transcripts individually, focusing on narrative content in order to get to know the data in all the details and nuances. During this reading of narratives, the researchers made detailed notes highlighting potentially important phrases and writing some explanatory and personal interpretative comments. These ideas were named where possible and coded.

In the second stage the data codes were organized into higher conceptual level entities referred to as categories. The main categories were detected by coding discrete passages of transcribed narratives. The two researchers who did the interviews independently prepared two lists of preliminary categories. Using extended interview passages to support their coding, these researchers discussed their preliminary codes with the third member of the project team, whose role was to verify and to critique the developing ideas. The slightly different lists of codes and their interpretations made by the two interviewing researchers and the third researcher were reconciled and sorted into categories. The categories were named based on the substance of the codes they were made of and the subsequent idea.

The third stage began as categories and themes were identified, discussed, debated, and refined. The professionals who participated in this study all had come into contact with a range of different experiences in caring for people who were dying, most of whom were middle-aged or older. A number of interviewees offered detailed statements of a compelling experience that might have become a theme, but that was not developed by many other participants. Most of the results of the study are constructed from the participants’ accounts of specific experiences blended with their more general points and emphases Participants discussed many examples in which their ideas about dignity were presented in terms of their opposites, clinical cases in which there were difficulties that threatened or denied dignity. 

From these complex accounts, many initial categories were identified, but the majority of them did not achieve a high degree of consistency across the analytic process. For example, the complications concerning expectations for the exchange of money for services was forcefully presented as a threat to patient dignity, but it did not become a theme because it was not developed enough across interviews with professionals to merit recognition as a theme. 

At a certain point, it became clear that listing themes, even some new and unique Lithuanian ones, was inadequate as the way to convey the central element or essence of what participants’ narratives were about. Almost simultaneously, the researchers realized that they were developing yet another list of important elements of dignity, but not fully conceptualizing something more essential that the participants were saying collectively. After a great deal of discussion and reexamination of both themes and categories, the researchers agreed that the essential theme that permeated what participants were saying is that a core component of dignity is “Being Heard”. The team furthered this understanding through dialog and reexamination of project data. The results are presented below. 

The aim of the fourth stage of data analysis was to ensure that themes correctly represented the empirical data. In order to ensure the trustworthiness of the findings the third member of the team independently reviewed corresponding data. The researchers discussed what exactly they meant by each theme and how every theme helps to understand (reveal) the aim of the research, the nature of dignity and how it can be enhanced. While the researchers held only slightly different suggestions concerning names of categories and themes, they easily agreed upon the meaning of them and the reconciliation of the final list of themes was made with a high degree of concordance. The last stage was writing up the analysis of the data.

## 3. Results

In seeking to understand the phenomenological essence of dignity at the end-of-life as perceived by professionals who provide care, four themes recurred with sufficient frequency and effect to become primary themes. The primary themes are Physical Comfort, Place of Care and Death, Effects of Death as a Taboo Topic, and Social Relations and Communication. In addition, an overarching theme constituting a core part of the essence of dignity at the end-of-life was identified. The essential theme of Being Heard knits together the four primary themes and extends them to a very fundamental aspect of dignity.

### 3.1. Physical Comfort 

Physical Comfort involves a state of peace, with effective symptom management, including pain control and minimal distress. Physical comfort was seen by participants as a necessary component of dignity but not as a problem, reflecting their faith in the medical means available to manage pain. One participant expressed faith in institutional care to provide pain control.

*I think first that there should be no pain. This is essential. In institutions, patients do not have this problem. In this sense, everything is organized and managed perfectly. Sure, they are anesthetized as they individually need. Not only strongly following written instructions, meaning that does not matter—If the patient is in very big pain or not, she/he will get drugs at indicated time only. We relieve pain as needed. Patients don’t have such problems. (nurse 1)*

An important part of this statement about pain control is that “This is essential”. Similarly, another participant stressed the centrality that pain control represents in patient dignity.

*Relief of pain is the most important thing. Of course. But contemporary medicine has means to relieve pain effectively. (physician 1)*

Along with pain management, cleanliness of the patient’s body is an element of comfort that contributes to dignity. Participants shared how maintaining hygiene is problematic in some cases in which the patient has a history of poorly met physiological needs, especially on the part of isolated persons or those with addictions that had overtaken self-care. There may be a clash between professionals’ knowledge of what a person might benefit from and what the individual wants or is motivated to do.

*Well, what else hinders a dignified death? Well, how the person treats his/her own body. How he has looked at his body from a young age and how tidy he keeps it. But sometimes you find... if a person has never taken care of himself, then in case of a serious illness dignity will be big problem. Because we find them not supporting body hygiene and laying in a messy environment, even though they are still capable of taking care of themselves. But it is their way of life. It seems normal to them. There are also alcohol addicts. Then their environment is also bad, for example, the roof of the room is dripping. And he almost dies. What kind of dignity is that? (nurse 2)*

Privacy and respect for one’s body is an important element of this aspect of dignity. Hospitals and nursing homes sometimes present problems in maintaining privacy, and thus promoting dignity, according to participants. For example, one participant integrated the indignity of a lack of respect for privacy with a recommendation for institutional personnel.

*There is a very easy answer here, in general, about the dignity of the patient, because here we understand that, for example, when climbing from one wheelchair to another, no one gives a cover and has to be naked all the way from that wheelchair. We can gather a lot of such tiny details, and how to solve it, the systematic answer to that, means we have to work with staff, it means investing in our human resources. If we teach and bother to look, always imagine that you are in the patient’s place or not, would you like to be treated like this or do it to you, or think that you are the patient what would you like to be done with you. (physician 2)*

### 3.2. Place of Care and Death

The theme Place of Care and Death concerns both the physical and social circumstances of death. It shares content with the theme of physical comfort and pain control.

*Dignified end of life is… [being] in a safe environment when basic needs are met… Eating, clean environment, warmth, comfort when you do not feel pain and when there are people around you... And that is the dignified end. When you can be calm and you know you will leave... But you will leave in peace. Not in chaos, not for someone. (physician 3)*

*The environment itself is important for dignity. It is important not to have poverty, to be above the poverty line. (physician 4)*

The professionals understand that patients typically want to die in the familiar surroundings of home, close to loved ones. They also recognize many patients’ aversion to death in an institution when there is no further help available there, as hospitals and nursing homes are currently organized.

*Oh, they do not want to go. He asks the child to “stop dragging me, let me die in my bed”. This is mostly the case. Well, he says, “What else will help me again?” So right, they don’t want to go to hospital. Very reluctant. If they are asked what they want and if they are conscious, they say that. (nurse 3)*

Spending the last days in the institution increases the likelihood of dying alone, which diminishes dignity when the patient does not want to be alone. Participants detailed their experiences with the difficulties of balancing the resources that may be available in the institutional environment with patient desire to be at home while maintaining patient dignity. Discussing and negotiating the place of care and death may involve relatives’ decisions to bring the ill person to the facility because they have difficulty in providing care, or difficulty with the idea of being with the dying person at the end of life. Failure to negotiate this situation can diminish dignity, especially when feelings and true motives are suppressed. 

### 3.3. Effects of Death as a Taboo Topic

The difficulties around the place of care and death are entwined with the third theme, Effects of Death as a Taboo Topic. Professionals understand the difficulties that many people have in communicating effectively about the end of life. The difficulty in openly discussing death occurs particularly often concerning its inevitability and where the death will ultimately occur. An aversion to discussing the impending death of a loved one or even an individual’s own death is common in participants’ experiences. Participants are familiar with relatives’ attempts to protect patients and themselves from directly facing the end of life.

*Relatives want to protect patients from bad news, but sometimes they make mistakes. Sometimes a [dying] person wants to know. And the relatives do not say that they are taking the person to a palliative care unit. They say that you “are in rehabilitation”. They do not invite a priest. (physician 4)*

Several participants acknowledged that circumstances and individuals varied. They did not assert that directly confronting the reality of a situation is essential to dignity at the end of life. In fact, they discussed how they and others sometimes avoided the topic. The category “giving unreal hopes for the terminally ill individual” that contributed to this theme was prominent in the stories shared by informants. Sometimes informants tried to “calm down” the dying person this way, seeing it as a sign of “humanity”. One nurse described the situation this way.

*We don’t say you are dying. We’re still giving some hope to the person. And many things we tell them. “Let’s wait for tomorrow, we will call your doctor, maybe she’ll prescribe something else”. You try to calm down the person all the time. After all, humanity is everywhere. It depends a lot on person. If he’s religious, he’s much calmer. (nurse 3)*

Denial, suppression, and fear of talking directly about the situation, for better or worse, are not, Lithuanian cultural phenomena. However, this constellation was expressed so widely as a characteristic of Lithuanian care situations that it becomes a significant aspect of the theme. The professional may have an important role in ensuring dignity through the ability to respect people’s existence within their culture, sometimes including and respecting its taboo. However, they also have the professional role of being honest in communing reality. Participants even recognized that care providers may share the cultural aversion to discussing death, which is sometimes seen in the form of the aloof clinician or the indifferent care provider. However, some participants clearly placed responsibility on the professional to find a way to confront the reality of the end of life.

*If a professional shows that he or she is ready to speak on the subject, the patient will always want to. A terminally ill person thinks about and feels the impending death subconsciously. I think a person’s anxiety and panic increases if there is nobody to speak to. (physician 3)*

### 3.4. Social Relations and Communication

Participants link dignity with Social Relations and Communication. This theme includes the significance for dignity of maintaining meaningful relationships with relatives, friends, and colleagues as well as medical professionals. Dignity depends in large part on the interactive communication of ideas and emotions among the patient and members his or her social circle. Support and sharing meaningful aspects of life are achieved through relationships. Tightly restricted access to medical facilities and even homes due to COVID-19 created particularly difficult situations for maintaining relationships. Like everyone else, priests were prohibited from having direct contact with patients in most settings. Others were afraid of infecting others or becoming infected themselves. This left patients unable to engage in the traditional and personally meaningful in-person spiritual conversations. Participants’ stories revealed the importance for dying people to see a priest in person. One or more visits by a priest is an old Lithuanian Christian tradition, even when the individual has not been an active Catholic. This is apparently especially important for elderly patients, many of whom were susceptible to Covid-19. In the run-up to death, a meeting with a priest becomes very important in maintaining or even achieving a state of dignity. As a nurse observed:

*Still, spiritual support is very important. They [dying people] ask for the priest. Perhaps many do not have that true faith, but I see that in the end, in the face of death, they want it. And then they seem to relax, calm down. (nurse 4)*

A nurse assistant discussed the problem of quarantine disrupting the possibility of spiritual relationship.

*[Patients] pray when they are left alone. Read. Read the Bible. Well, I am not present at the time when Mass is shown on TV, but they ask to turn it on in advance. They ask next time to bring the priest to make a confession, but... Now all year that quarantine. Before the quarantine I had to call, talk to the priest so he could come and stay and talk. And now that quarantine. (nurse assistant)*

The taboo concerning communications about life and death may be expressed through avoidance of Social Relations and Communication with the person facing the EOL. People who are averse to contact with the person with terminal illness are likely to be constrained by a lack of knowledge about how to communicate with a person approaching death, which leads to further narrowing of the social circle. Professionals found that patients are often limited to social relations with care providers and close relatives, leaving out others who may be important to them. This situation may lead to a sense of diminished worth and dignity. 

Professionals are in relationships with patients and families, whether they are aware of the fact and its potential importance or not. Difficulty in mindfully making use of these relationships and knowing how to communicate to enhance dignity was often cited as an area of concern in need of improvement. One nurse suggested that a specialist should be dedicated to these communications, simply stating that “*A psychologist is needed to help the patient and relatives and staff communicate*”.

Participants often noted that basic professional education and continuing professional education should include developing the ability to talk about the patient’s and family’s situation.

*We’re not ready to talk to patients about death, and we see that we have something to do with it... no one teaches or prepares for it. (social worker 1)*

### 3.5. Being Heard, an Essential Component of Dignity

The fifth theme is Being Heard. This theme is of a higher order than the primary four in that it contains elements of each of the primary four themes and goes beyond them in significance. Many participants stated in one way or another that to patients Being Heard constitutes an essential component of dignity at the end of life. As a theme, Being Heard means being able to express oneself and the acknowledgement that one’s expression is listened to and taken seriously. The professional caregivers sampled recognize that insofar as possible, meeting the terminally ill person’s will and wishes is a critical element of dignity at the end of life. This theme involves both small, everyday wishes and major life choices. Hearing may involve participating in unpleasant, awkward, and unrealistic communications as well as communications that are positive and gratifying. Communications may have meaning to patients that are not obvious, and Being Heard means that someone has taken an interest and expresses the desire to understand, to hear meaning. This theme was made most apparent by numerous examples of its omission from the care provided. One participant detailed how family caregivers sometimes resisted the patient’s will to see a priest, stating for example that “Mother had never believed in God before”.

Examples of simple wishes include the patient’s desire to smoke or to be provided with specific food, which sometimes involves clinical decisions about medically ideal care versus experiences the patient wants. A particularly difficult element of this theme is found in the matter of mandatory resuscitation and other life-prolonging measures when extremely ill patients might not benefit from them or want them. Even when it is clear that an individual does not want to prolong suffering, mandatory resuscitation and other measures can threaten dignity.

Being heard also means that the patient’s expressions of his or her will are taken seriously and acknowledged, even if they cannot be fulfilled or accomplished. Both the expression and the acknowledgement by caregivers and others are essential. Hearing and acknowledging are deliberate parts of professional practice, according to practitioners.

*Every moment, every day, to hear a sick person, to listen, to perceive, to recognize his choices, and desires. Every day, every stay nearby to try to understand, to read his or her will. Each person should be seen individually. You (health care professional) have to be in an individual [patient’s] process... There is not any draft. (physician 5)*

Each of the four primary themes is linked with the essential theme of Being Heard. For example, a dying person’s expressed wishes often involve the place that they want to be during their illness and perhaps their death. Similarly, physical comfort and pain control can be either cooperative expression of will coupled with hearing and response, constituting a dignified process. If a person is seen as an object to be treated and manipulated without engagement of the will and wishes of the person, dignity is much less likely to be a component of the experience.

Relationships and communication are inseparable from the idea of Being Heard. Relationships are the ways that people express meaning to one another. They may involve both family and close friends and professionals, including clergy. Relationships imply communication, in which each party can express and be acknowledged. Participants noted that as the end of life nears, the circle of people in active relationships to a patient tends to shrink, and the importance of professionals working to maintain the patient’s important relationships was a frequently discussed interview category. Relationships involve communication, the interactive sharing of ideas, wishes, and emotions, all elements that must be dealt with effectively for dignity to prevail. Participants concisely presented the affirmative case for Being Heard as an essential part of dignity.

*Dignity is based on the ability to understand, to hear, the patient’s thinking, not to come with your own understanding of the situation and your own desire. It means to satisfy desires of the patient. It means to go in the direction where the patient himself is turning. (physician 5)*

*Everyone must have a sense of the other person where that person is. (physician 4)*

Being heard is unlikely occur when the unwritten cultural norm of avoiding the topic of death overcomes the ability to listen and communicate. The effects of the taboo around discussions of death are seen repeatedly by professionals. Patients, family members, institutional staff members, and professionals all are subject to the taboo in some uncomfortable circumstances. The effects of deliberately avoiding death-related topics of communication can be reflected when each of the other themes is not provided for. There is no corrective dynamic at work or available to professionals when the taboo is held to by anyone in the patient’s world. A sophisticated professional skill is needed when the patient’s will is to participate in the taboo of not talking about the EOL.

## 4. Discussion

The aim of this exploratory study was to understand attitudes, experiences, and suggestions of Lithuanian health care practitioners concerning dignity at the EOL. Thus, the researchers addressed this aim by using an interpretive, phenomenological approach. The research questions addressed were straightforward: How do Lithuanian health care professionals understand the essence of dignity at the EOL? How do they believe that dignity at the EOL can be enhanced? How do professionals understand the essence of dignity?

Most studies of EOL dignity look at the topic through the lens of empirically developed survey tools. Overall, the current study’s findings are consistent with most elements of other studies and with the Dignity Model developed by Chochinov et al. [9]. The current study was intended to determine the essence of how professionals see patient dignity and of how it can be enhanced. The study was framed from a different, phenomenological viewpoint. In phenomenological research, the essence of a phenomenon is a defining characteristic that captures critical aspects of the phenomenon. The study involved ascertaining the essence of something that is experienced by people in different ways, and it sought to synthesize a common conception of the central characteristic of the phenomenon of death with dignity. In this study, professionals’ accounts revolved around Being Heard, making it an essential component of dignity at the EOL. 

Being heard obviously has limitations and different meanings depending on an individual’s condition, history, and beliefs at the EOL. No two deaths are identical, and generalisations about the phenomenon of dignity at the end of life must be understood in both individual and cultural contexts. For example, studies of Romanian, Iranian, and Lithuanian nurses found that these professionals reported that spiritual guidance or participation was particularly important to dignity [14,21,22,37]. In the current study, spirituality was noted by some participants, but it was not discussed frequently enough or specifically enough to be considered as a theme on its own. However, it is quite consistent with the themes involving relationships and communication, taboo, and place of death. That is how some participants presented the topic.

Being heard is primarily a matter of being acknowledged and valued as a person. Being heard is an expression with a number of elements from the primary four themes. Of particular significance is the theme of Social Relations and Communication, meaning the processes and emotional connections between people. Being heard involves not only being able to express a view, a concern, a question, or a request. 

Being heard means that one is acknowledged as a person worthy of being listened to seriously. In clinical settings it might mean that the patient is free to speak or convey something through body language, even if it seems trivial on the surface. Being heard also means that someone who hears has been open in a fundamental relational way to what a dying person wants and feels. The patient’s will is not only a simple wish or preference, but instead is a deeply felt wish or appreciation for something important to that person. It may be a desire for reassurance about going to heaven rather than hell, or staying at home rather than being taken to an institution. It may be a desire for a specific sort of comfort and pain care or family visiting. And it may be a desire, whether acknowledged or not, simply to be listened to or talked with on whatever topic may be at hand, for example when elderly patients recount significant life experiences. It may require communication with someone with the ability to do something, even if it is just to listen. Being heard encompasses many of these conditions in the Lithuanian context, and professionals report that it is not limited to people who can verbalize their will at the EOL. It also extends to expressions of their wishes and feelings before they are incapacitated, particularly because formal preparations of wills and living wills is still uncommon. Professionals reported that Being Heard is not the same as the content of the communication. It is a process of valuing through communication. For example, sometimes these communications start with requests for spiritual guidance, and sometimes they may be requests for something very practical. Being Heard includes taking in and acknowledging communications beyond literal meaning of words, particularly in expressions of emotion.

How can dignity be enhanced?

The importance of communication is recognized within and between organisations, not just between professionals, patients, and relatives. Lithuanian regulations, laws, and some practice protocols are basically similar to those in countries that have established highly functional organisational and practice principles [38]. There are examples of excellent care honoring and promoting patient dignity at the EOL in Lithuania. However, Lithuanian professionals have implemented relatively little in the way of coherent philosophies, models, and best practices for this kind of care. 

The principles of a clear and public philosophy and approach are well represented in the sphere of hospice care as conceptualized in other industrialized countries. One key element of these approaches is teamwork, including professionals supporting one another in hearing and understanding a person at the EOL. This type of care is only beginning to be implemented in Lithuania. However, the participants in this study stated a desire for using a number of ideas about dignity-enhancing practice that are consistent with these successful models. Sometimes these ideas were directly stated, and sometimes they were presented tacitly through examples of inadequate practice. It is noteworthy that ideas that were presented by Lithuanian professionals about best practice are consistent with those contained in guidelines from other countries. For example, Hospice UK simply states what they aim to do: “Hospice care looks after someone’s physical, emotional, social and spiritual needs. This means that hospices provide a wide range of services” [39]. Each of these facets of hospice care is addressed in the professional’s accounts concerning patient dignity.

One impediment to progress appears to be the avoidance of discussing death either in the general culture or in the specific context of individual patients and families. The theme of Effects of Taboo may be exaggerated in Lithuanian culture, but it is not exclusively Lithuanian. For example, difficulty of discussing important matters at the end of life, particularly listening and hearing the dying person, is so widespread in the United Kingdom that another British organisation, Dying Matters, specializes in “Talking about death and dying”. They aim to make public the idea that talking about death is possible and desirable, offering, for example, a practical document entitled “What to say, how to say it and where to find help” [40]. In the United States the Institute of Medicine compiled the work of numerous experts in a major volume entitled *Dying in America: Improving Quality and* *Honoring Individual Preferences Near the End of Life* [41]. The volume includes many empirically supported recommendations for palliative and hospice care, notably emphasizing “honoring individual preferences,” and detailing how professionals, families, and patients can be engaged in this process. These are examples of models for reform in EOL care that is very consistent with the essential theme of people Being Heard at the end of their lives.

### Limitations and Implications for Further Research

The existing body of research makes it clear that there has been little research on dignity at the EOL as perceived by professionals in different national and cultural contexts. This study represents a prelude to a program of research on the phenomenon of dignity and on practice to preserve and enhance it. Future research can systematically use this foundation and examine the findings with more precision and control of the limitations of this study. The strength of the phenomenological method is that it can lead to understanding “from the inside-out” with relatively little pre-determined structure of the data. The participants share information from their own, in this case, Lithuanian, context and experience. However, this strength of the method means that it can be highly depending on this context. For example, the study occurred at a point of high incidence of COVID-19 and the concomitant hospitalisations and deaths. Many of these deaths occurred while patients were necessarily isolated in conditions that allowed little or no communication of important thoughts and wishes that might otherwise be heard. Perhaps the inability to communicate affected the participants’ recognition of the significance of Being Heard. It is noteworthy that COVID-19 seldom came up in the interviews, and the relationship between COVID-19 and dignity is an area ready for exploration. 

The findings of this and other phenomenological studies set the stage for follow-ups using different methods. The same is true for the surveys that are so prevalent in the literature. In particular, study of patients and of actual care *in vivo,* is warranted to understand the communication and relational processes involved in Being Heard, negotiating the “truth” with patients who live in a culture where discussion of death is practically taboo, and negotiating where people will be cared for. It is also unclear whether the use of audiovisual media for interviews affected the findings. Understanding dignity ultimately will require involving patients who are at the end of their lives in future studies. Of special significance is the importance of considering the influence of patients’ life stages at the ends of their lives, especially considering the distinctive characteristics and needs of geriatric patients.

## 5. Conclusions

The practitioners who offered their understanding and experience to this study have provided a foundation for the next phase of the process of enhancing dignity at EOL in Lithuania. This process involves shifts in culture, here meaning a nation’s widely understood and enacted attitudes and ways of doing things. Consistent with the Pringle et al. [9] finding that a systematized approach to care was positively related to the maintenance of dignity, a Lithuanian body of knowledge about how practice affects dignity is emerging. Medical professionals and citizens have begun to show some cultural consensus and a commitment to reforming problematic areas and to enhancing the principles of dignity that study participants have shared. These efforts are not yet widespread. The lingering influences of rigid bureaucratic thinking and dehumanizing Soviet occupation on contemporary medical and popular culture must be assessed continuously, especially as they affect the aged. The participants in this study understand something important, and they appear to represent a will to enact coherent policies and models of practices to enhance dignity.

The themes of this study are interrelated. The findings are consistent with those of Guo and Jacelon and Sylva et al., but the Lithuanian participants provided a different emphasis. The primary finding is that patients are likely to have something to express at the EOL, and professionals can enhance dignity by sincerely hearing and responding. This finding might not be so significant if participants did not identify it so often by its absence. Hearing patients in this sense can occur in the course of dealing with pain or planning or adjusting to institutional care, both themes shared by the practitioners in the study. Practitioners can enhance dignity by learning sophisticated skills concerning communicating and relating with patients, and this is particularly important in EOL situations. Excellent resources are available. For example, Pino et al. developed a sophisticated method for engagement with terminally ill patients whereby they are not directly asked what they want or are concerned about, since it has been shown that this can be off-putting and counterproductive [42]. Instead, Pino et al. offer a way to engage with patients in which the professional learns to hear cues and clues that can be developed further in natural discussion. The key is that patients have to be able to engage in a process of communication in which they are heard and acknowledged, taken seriously. Practice milieux can support or discourage this sort of relationship and communication. As Pringle et al. [13] noted, this kind of interaction is most likely to occur when there is an organized practice approach with articulated values and ways of work that emphasize patient dignity. Patient dignity is both a human right and a constitutional right in Lithuania, but in many settings, it remains an aspiration rather than a reality. Building the question “Is this patient being heard?” into practice protocols and conventions would be a step toward the aspiration of enhancing dignity at the EOL.

## Data Availability

The data supporting reported results can be found at the Department of Bioethics, Lithuanian University of Health Sciences. Send request to ruta.butkeviciene@lsmuni.lt.

## Supplementary Materials

The following are available online at https://www.mdpi.com/article/10.3390/medicina57121318/s1, File S1: Interview Guide.
