# Supplementary material for: Being Heard: A Qualitative Study of Lithuanian Health Care Professionals’ Perceptions of Dignity at the End-of-Life
Source: Medicina (Kaunas). 2021 Dec 1;57(12):1318. doi: 10.3390/medicina57121318 (PMC8707950; doi:10.3390/medicina57121318)
Supplement: Supplementary file 1 [file medicina-57-01318-s001.zip › medicina-1445962-supplementary.pdf]

INTERVIEW GUIDE

For professionals working with patients with severe and incurable diseases

- What needs, according to your professional experience, are the most important for terminally ill persons and their family members at the end of ill person's life?
- What challenges and difficulties did you experience in meeting the ill person‘s needs?
- Who and how usually participate in making treatment and care decisions?
- How do you personally understand dignified end-of-life?
- What factors, according to your professional experience, ensure dignity at the end of life?
- What are the barriers to ensuring a dignified end-of-life for terminally ill persons?
